# State-society nexus in Brazil and Venezuela and its effect on participatory governance efforts in health and other sectors

**DOI:** 10.1186/s12939-020-01278-1

**Published:** 2020-10-26

**Authors:** Qamar Mahmood, Carles Muntaner

**Affiliations:** 1 International Development Research Centre, Ottawa, Canada; 2 Collaborative Program in Global Health, Bloomberg Faculty of Nursing, University of Toronto, Toronto, Canada

**Keywords:** Participatory governance, Latin America, Health inequalities, Brazil, Venezuela

## Abstract

**Introduction:**

Participatory governance is about state and society jointly responsible for political decisions and services. The origins and trajectory of participatory governance initiatives are determined by the socio-political context and specifically the nature of state-society relations. Participation by communities in health interventions has been promoted globally as a strategy to involve citizens in health decision-making but with little success. Such participatory governance in health should be seen not as a strategy alone but as a political project in which organized communities challenge the status-quo in health.

**Methods:**

This paper deals with the wider socio-political context of participatory governance initiatives. It uses comparative politics literature to analyze socio-political context in Brazil and Venezuela, historically spanning half century prior to 2015, to assess whether it was conducive to participatory governance. The focus of this paper’s analysis particularly is on the socio-political changes that were taking place in Brazil and Venezuela in the decades of the 1980s and 1990s. Those decades formed the bedrock on which the two countries experienced democratization and a socialist transformation that has lasted well into the first decade of the twenty-first century. The situation in the health sector is also described for the two countries showing a parallel trajectory to the wider political context and that reflected the political ideology. For this assessment, we use a contemporary framework called the ‘socialist compass’ which links dynamics of power relations in various ways among three domains of power, namely, state power, economic power, and social power. Socialist compass can be used to assess whether such reforms are moving towards or against social empowerment.

**Conclusion:**

Our analysis reveals that both Brazil and Venezuela were moving in the direction of social empowerment until at least the year 2015, just before the political turmoil started engulfing the left-leaning regimes in both the countries.

## Introduction

This paper argues that the socio-political context, specifically state-society relations, affects participatory governance initiatives or reform processes in health and other social sectors. Numerous reform processes in health have focused on community participation to improve health with the goal of achieving Health for All (HfA) and ensure greater equity in health. Ideologically, participation has different meaning for different actors in the society. From a leftist point of view, it is about reconstruction of a broader concept of democracy [1], while for international development agencies, participation by communities in reform process promotes accountability [2]. Participation by communities is seen as a strategy towards achieving such goals to improve health. What is not realized is that it is more than just a strategy that ensures that communities participate in state affairs, such as in the health sector [3, 4]. Organized communities participating in political decision-making is about participatory governance to achieve social goals and democratic participation in state affairs [5]. As such, it is inherently political in nature. Participation by communities should therefore be seen in the wider socio-political context. This paper looks at the wider socio-political context and specifically the state-society relations in how it impacts the origins and trajectory of participatory governance initiatives.

The aim of this paper is to explore the origin and trajectory of participatory governance initiatives in health, and more generally, by situating them in the wider socio-political context in two Latin American countries of Brazil and Venezuela. We argue that these countries in particular were at the forefront of democratization and socialist transformation that the continent experienced in the late twentieth century and the early twenty-first century. For this comparative politics country analysis, we use an analytical framework that identifies state-society relations at its core and how those relations are shaped by the interplay of power dynamics among state, society, and economic powers. The analytical framework, known as ‘the socialist compass’ [6], interestingly is not static but is dynamic such that it indicates movement towards or away from social empowerment of reform processes such as participatory governance initiatives. We first provide some background in the paper before introducing ‘the socialist compass’. This is followed by the two country cases mainly based on literature, relating to health and more broadly in the fields of political sociology and politics, from scientific, peer-reviewed publications, policy documents both nationally and internationally, as well as grey literature. Lastly, we use the framework to analyze the country cases and draw conclusions and policy recommendations.

We believe this paper provides a unique analysis, at least in the field of health, by highlighting the socio-political context and specifically the state-society nexus in looking at health reform processes which is the strength of this paper. While the analysis of this paper is a strength, the two country reform processes also offer unique perspectives. In both Brazil and Venezuela, the reform processes in health were at the forefront of and harbingers of the democratic transformation that was taking place in those countries. The health reform processes were setting the groundwork for other sectors and the wider society to follow in this socialist transformation. The limitation of this paper is that for a topic so deep and extending over a period of several years, it is challenging to capture that in one paper. The other challenge is to carry out comparison of two diverse countries, which, while they have several contextual similarities, are also quite different. To overcome these challenges, we have used comparative politics literature on these countries, includingliterature that speaks to the contextual realities of the Latin America, and a contemporary analytical framework against which we have looked at the country cases to ensure uniformity across the two contexts.

## Background

Achieving universal and equitable population health requires involvement of both the state and society. Health has been recognized as a fundamental human right. State has the prime responsibility in guaranteeing this right. The Alma Ata declaration in 1978 recognized that people, individually and as organized citizens, have a duty to collectively participate in the planning and implementation of activities to ensure the realization of their right to health [7]. Numerous international declarations in health such as HfA, Primary health care, the Ottawa Charter, and the World Health Organization (WHO) report of 2008 ‘Primary Health Care: Now more than ever’ [8] as well as the Commission for Social Determinants of Health report [9] have emphasized the importance of achieving population health equitably while recognizing the vital role that states and communities/civil society can play.

Governance issues involving the state and civil society have gained prominence inachieving equitable health development [10, 11]. Whereas there has been an increased emphasis on the role that civil society can play in participatory governance, it has often been at the expense of a diminished state. Reforms, such as decentralization, have been implemented to bring services and decision-making processes closer to the communities [5]. Such reforms have aimed to make states either more efficient or more democratic. The efficiency aim has been predominant and was mostly conceived in the neoliberal ideology [12]. The predominant understanding of the idea of civil society has also been in an individualistic and liberal ideology, putting paramount importance on civil society autonomy in its role to monitor the state and political society [13]. Conceived in this way, the role of civil society is seen as putting external pressure on the state. Consequently, a rigid dichotomy between state and civil society is created. While civil society autonomy is vital for civic organizations to not get co-opted by the state, an increased emphasis on civil society autonomy undercuts the idea of a synergy between state and society that can be important for participatory governance. Diminished state, neoliberal economics, and empowering of liberal/individualistic orientation of civil society at the expense of civil society that is organized around communitarian principles has created democratic deficit.

While the causes of this democratic deficit have been broadly the same across developed and developing countries, the effects have been different for the two. The latter has been especially hit hard because of their already exiting vast social inequalities and deep social exclusion [5]. Elite democracy and neoliberal policies played havoc to the already existing social cleavages in these countries. On the one hand electoral representation, or a system of competitive representation based on elite democracy, has restricted mass participation consequently limiting representation of civic interests in the state [14]. On the other hand, implementation of neoliberal economic policies has promoted privatization and deregulation of the economy and diminished the role of the state [15]. The consequences of such policies have been strengthening of the interests of business/corporate classes while labour and working classes have been weakened further. Thus, an elitist democratic political system and a neoliberal economic system together have widened this class divide resulting in worsening of inequalities [16].

Social inequalities resulting from elitist democracy and neoliberalism have alienated the majority while a minority elite strengthened their grip both politically and economically [17, 18]. Whether it was authoritarian regimes in control of state power, or later, when such regimes were over-thrown during democratic transitions in most third-wave democracies, the interests of this elite group never ceased to be represented through state power [19]. Social exclusion of the majority from political and economic spheres gave rise to civil strife, seen in many third-wave democracies such as those in Latin America [20]. At the same time, this alienation of the masses inculcated a search for alternatives which was led by a variety of social movements that comprised of groups that were politically and socially excluded [21, 22]. These alternatives emerged as democratic societal practices that abound in many third-wave democracies of Latin America, arising in response to the repression of autocratic political regimes, and continued to flourish when political society in post-autocratic era continued to show signs of non-democratic practices in many such states [23]. However, despite the undemocratic practices of political society and the numerous examples of democratic practices at the societal level, democracies continue to be assessed in their political sphere only, essentially meaning holding of elections every few years [24]. Further democratic consolidation in both developed and developing countries, especially in the latter (which includes mostly third-wave democracies), cannot be achieved unless democratic practices at the societal level are harnessed and institutionalized [17].

Heller [5] has described state-society engagement that is determined by the nature of local democracy and the political opportunity structures that the state provides for social movements to interact with it. Local democracy in the context of democratic deepening expands the surface area of the state and provides greater opportunity for civil society to interact with the state. The political opportunity structure for social movements relate to the power and control that can beexerted in determining what policy agendas or issues, political claims, and identities enter the political domain. Local democracy and political opportunity structures are interrelated because they are about the institutions and processes that are in place for the state and civil society to meaningfully engage. They are about the public sphere and how that is shaped by the political or economic power or by the deliberation of reason-bearing citizens.

## Participatory governance

The necessity of state-society synergy to tackle inequities is important. Understanding interactions between state and society or governance issues are important to address the inequity and injustice that are at the core of many health problems. Historically, empowered and organized groups and citizens have played a role in creating healthy social change [25]. Healthy governance aiming at health equity is participatory governance and it places emphasis on inclusion of people, especially the poor [10]. Important to understand in this sense is how citizens collectively organize and participate in state affairs? What are the opportunities that the state provides or barriers it creates that either promote civic organization and public participation or dissuades action by civic groups?

State-society relations present a contentious terrain to analyze. Participatory governance analyses involve societal and political levels. Analyzing sectoral implications in tandem with the societal and political aspects of reforms and interventions, for example in health, can be challenging but important. Often the way sectoral interventions are conceived, structured, and implemented ideologically reflect the underlying nature of participatory governance structures and mechanisms. With this hypothesis, our paper’s aim is to analyze the changing nature of participatory governance over time and the ideological alignment in the health sector. As mentioned above, this can be a challenging analysis. However, this paper will build on our previous work in this area in Latin America [26–28] and contribute to it by showing ideological alignment between health sector reforms in the context of wider participatory governance.

For this analysis, and particularly the specificities of the Latin American context, we looked at the following three theoretical/analytic frameworks that could provide a uniform and structured way for our analysis. 1) Leonardo Avritzer’s Public Space theory of democracy [17]. 2) Rebecca Abers’ Dilemmas of Participation [29]. 3) Erik Olin Wright’s Socialist Compass [6]. Avritzer’s theoretical framework strives to improve democratic practices by focusing on democratic societal practices rather than focusing on the political sphere only. He contends that public level democratic practices, which he refers to as ‘participatory publics’, need institutionalization. Public sphere is central to his theory which he describes by its location between the market and the state which he develops conceptually using empirical cases of societal practice from Latin America. Abers’ framework of Dilemmas of participation is built around the interplay of implementation, co-optation, and inequality leading to various levels of synergy between state and society. Other critical elements in this interplay include political strategy helping or hindering civic autonomy, and organized citizenry that can foster or impinge on the social inequality. Abers’ theoretical framework is squarely conceived within and used to analyse participatory governance experiences particularly in Latin America, for example the participatory budgeting experience in Porto Alegre.

While all three frameworks are useful for our analysis in certain aspects, we found Wright’s Socialist Compass to be most relevant and describe it below. Avritzer’s framework tangentially touches on the economic aspects by referring to location of the Public Sphere between the state and the market. However, he does not develop that more explicitly. Also, he lays out the idea of Participatory Publics which highlights the societal aspect, but the political aspects are not strong for our analytic purposes. Lastly, the framework is stronger on the theoretical side and some implementation on-the-ground aspects, but our focus is in between these two levels. Abers’ framework is very strong on the implementation side and at some level remains focused there. The interplay of state and society are strong but the economic aspects, such as Arvitzer’s reference to the ‘market’ is much less defined. Wright’s Socialist Compass presents the right mix focusing equally on the economic aspects in determining the level of synergy between state and society. Also, while conceptual in its orientation, the Socialist Compass, provides practical models depicting varying nature of participatory governance in the power interplay among the state, market, and society. The pathways of the Socialist Compass, in that sense provide the middle level focus between the conceptual/theoretical level and the implementation/on-the-ground experiences. We describe Wright’s Socialist Compass below.

## Three domains of power at the nexus of state and society

**Fig. 1:**
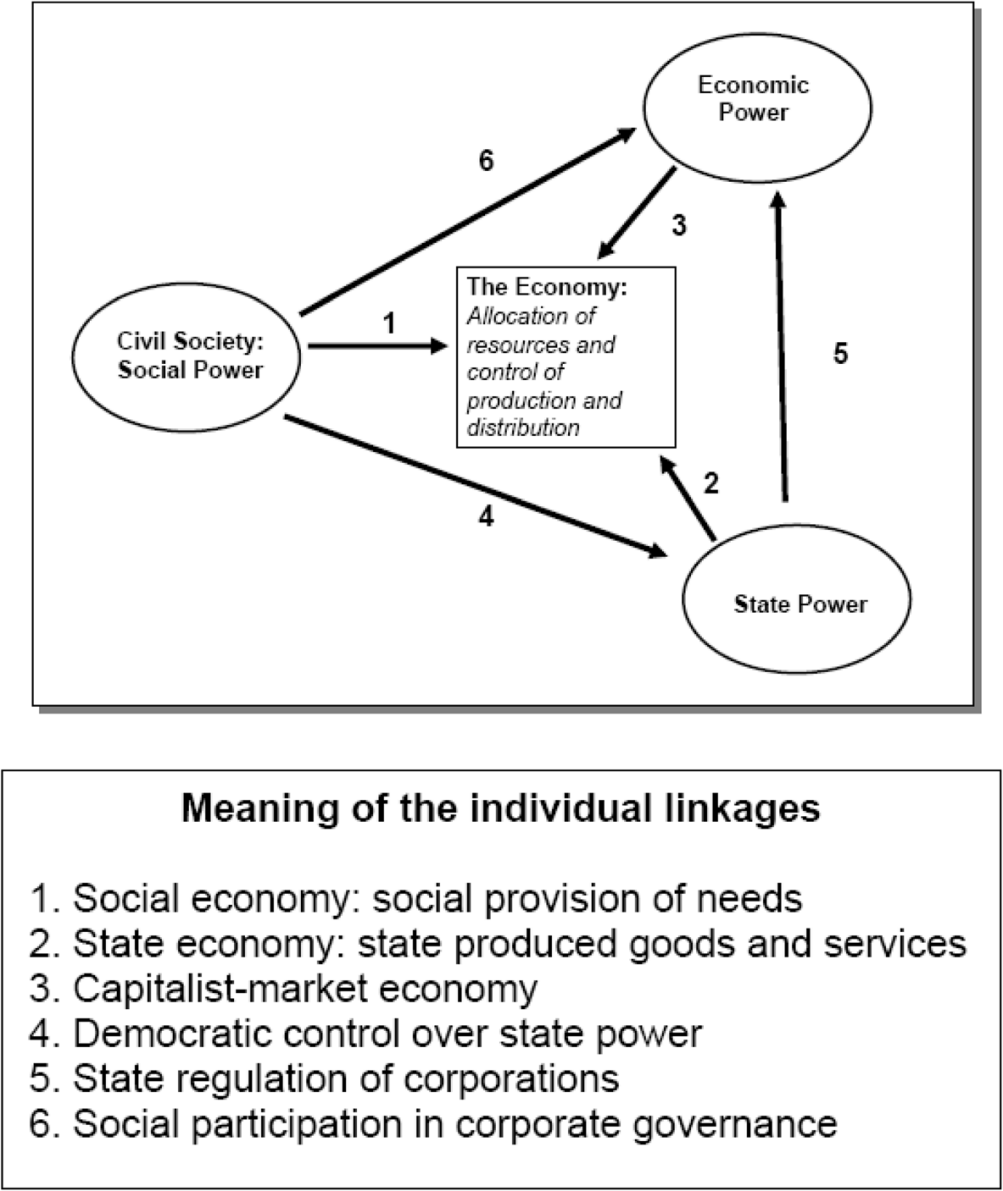
Linkages in the Pathways to Social Empowerment [14]

Erik Olin Wright [6], one of the foremost political sociologist has extensively worked on state-society dynamics. In his seminal work in 2010 he identifies three social domains of power, that operate on economic activity (Fig. 1). Economic activity, at its core is about the allocation of resources and the control of production and distribution of those resource. The three social domains of power are state power, economic power, and social power. The principal form of power that shapes economic activity determines which of the three power domains dominate. Whether it is state power, economic power, or social power that dominates will result in statist, capitalist, or socialist systems respectively. Wright acknowledges that historical experience with capitalism and statism has meant that examples of institutional arrangements for an economic structure based on these two systems exist. However, the same is not true for a socialist system in which social/civic power is the central organizing principle of the economy.

With this conceptualization of the three social domains of power, Wright suggests that the idea of democracy can be a way of linking social power and state power. Complete subordination of the state to social power would be ideal democracy. Social power in this sense, however, should not be “rule by the atomized aggregation of the separate individuals of the society” [6] but it is individuals organized in collectivities or civic associations such as parties, communities, unions, or worker cooperatives. In this sense democracy encompasses all spheres of life. Wright asserts that in this sense democracy is “… inherently, a deeply socialist principle.” [6]. If “Democracy” is subordination of state power to social power, then “Socialism” is the subordination of economic power to social power.

Wright mentions that this conceptualization of socialism, and in turn democracy, is different from what is traditionally understood by these terms. In this conceptualization, social power is the central element of power and in that sense social empowerment relates to power that organized civic groups have. Traditionally, socialism has been closely associated with statism, where the state is the central element of the control of a system of production and distribution. However, for this paper, this understanding of socialism will be limited to social empowerment as a relative interplay between social power and state power.

If social empowerment is about the relative power of organized civic groups or associations or social power to that of state power, then state-society interactions are key especially in terms of control and power over decision-making to achieve social goals. In other words, state-society interaction to achieve social goals is about governance issues. The relative strength of social power over state power will determine to what extent governance is participatory in nature. The trajectory of participatory governance initiatives would in this sense depend upon the socio-political context as determined by the nature of state-society interaction.

The context of participatory governance initiatives may be understood as interplay of power relations among the three social domains of power that Wright mentions and their influence on economic activity. Participatory governance strategies while empowering and participatory on their own, need to be considered in their socio-political context to assess their effectiveness. If social empowerment is understood as how much social power subordinates state power or economic power, then truly democratic and socialist systems should be the most socially empowering. However, most examples of participatory governance initiatives are based in societal relations that are highly capitalistic in nature. For Wright, in “the absence of a comprehensive institutional design for a radical democratic egalitarian alternative to capitalism …” [6], there is a need to identify principles of institutional innovation and change that can guide any effort leading to social empowerment, thereby indicating a move in the right direction.

## Pathways to social empowerment

In that context, Wright [6] devises pathways to social empowerment across the three social domains of power, as shown in Fig. 1, which he calls a ‘socialist compass’. The significance of calling it a ‘compass’ is to emphasize that it is not “… to propose blueprints for realizing the ideal of social empowerment over economic activity, but rather to elaborate a set of principles which tell us when we are moving in the right directions.” [6]. The figure is pictorial depiction of Wright’s ‘socialist compass.’ It shows the three social domains of power centered on ‘the economy’ and the linkages among them. Combination of various linkages in certain ways produces various types of social empowerment. Social empowerment can either operate directly over economic activity, over the way state power affects economic activity, or over the way economic power shapes economic activity. Arrows 1–6 in the figure “… represent effects of power from one social domain on the other and the effects of power directly on economic activities in the economy” [6].

**Fig. 2:**
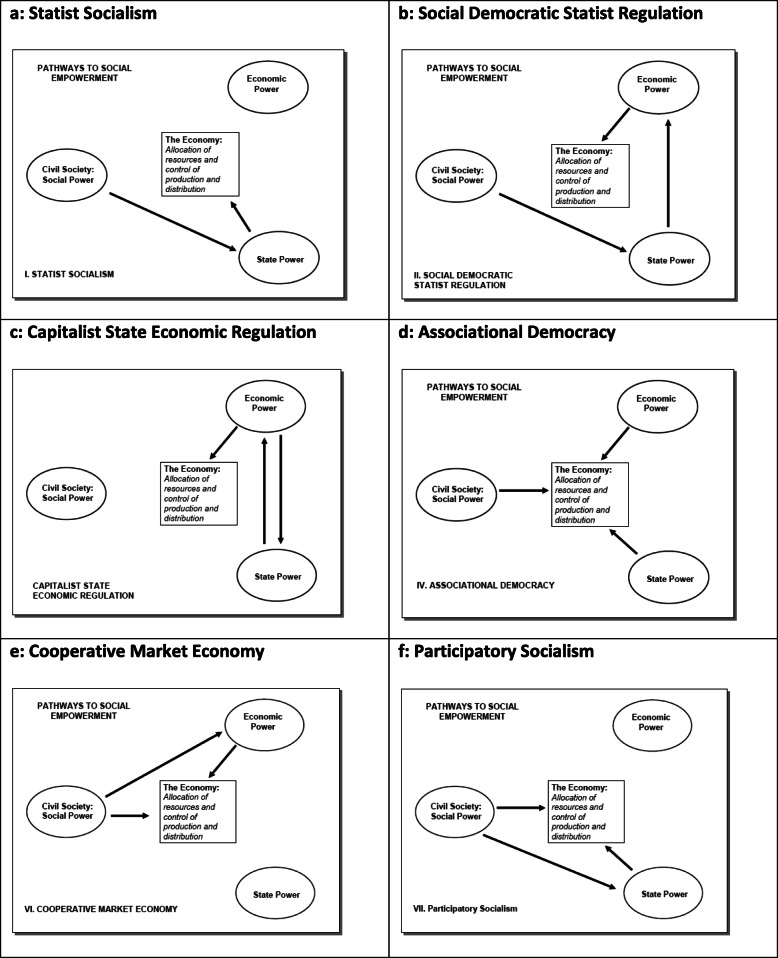
Pictorial depiction of pathways to social empowerment [14]

These linkages can then be combined into a variety of different configurations through which social power – power rooted in civil society – affects the allocation of resources and the control of production and distribution in the economy. Wright does mention specifically that the figure with its social domains of power and the linkages through which power is exercised only refer to social power and is not a comprehensive map of all power relations over economic activity. He then goes on to devise pathways that link these three social domains of power in various ways, Fig. 2 (a – f) for pictorial depiction and Table 1 for brief description of each, and how social empowerment can be achieved in each of these configurations.

**Table 1:** Pathways to Social Empowerment [14]

**Statist Socialism** Fig. 2a – The state remains a provider of many public goods that can benefit the working classes and the marginalized groups. For social empowerment, the state needs to be under democratically empowered civil society.	
**Social Democratic Statist Economic Regulation** Fig. 2b – One of the main functions of the state is its regulatory capacity. States in such cases thus act to put restrictions on owners of capital thereby keeping a check on economic power. Social empowerment in this case depends on what affect social power can have on the state through democratic political processes.	
**Capitalist State Economic Regulation** Fig. 2c – Regardless of how much influence social power can wield on the economy through democratic political processes on the state, it cannot be denied that capital has far more influence on the state. In capitalists’ societies, much of the state’s economic regulatory function yields to the needs of capital or economic power than social power.	
**Associational Democracy** Fig. 2d – An institutional arrangement that is coordinated joint effort of the three social domains. Health councils involving medical associations, community organizations, and public health officials’ in various aspects of healthcare are examples of associational democracy. Social empowerment results from such councils being internally democratic, representing broad interests of civil society, and undertaking decision-making through deliberation.	
**Cooperative market economy** Fig. 2e – This pathway deals predominantly with the economic domain and describes the nature of a system in which there are worker-owned cooperatives. Unless they are embedded within cooperative market economy, worker-owned cooperatives are disadvantaged as they are inherently socialistic but mostly based in capitalist economic structure. Cooperative market economy is oriented around cooperatives or based on such principles.	
**Participatory socialism** Fig. 2f – is a combination of statist socialism and empowered participation where there is joint involvement of the state and civil society in organizing and controlling various kinds of production of goods and services. In this case, state involvement is far greater than state’s role in social economy – social economy meaning voluntary associations of civil society directly organizing various aspects of economic activity. Similarly, social power is also greater in participatory socialism than statist socialism.	

In outlining these principles of the socialist compass, Wright mentions that social empowerment may partially be achieved if movement is restricted along individual pathways. However, substantial movement along several of the pathways is required for social empowerment that challenges capitalistic relations in a society. Lastly, he mentions three conditions that need to be met for the potential of social empowerment along the pathways described to be actualized. “First, it depends upon the extent to which civil society itself is a vibrant domain of collective association and action with sufficient coherence to effectively shape state power and economic power … Second, effective social empowerment depends upon the presence of institutional mechanisms which facilitate the mobilization and deployment of social power along these routes … And third, it depends upon the capacity to counter the deployment of power opposed to social empowerment. Above all, in the context of capitalist society, this means countering the power of capital as well as those aspects of state power opposed to initiatives and action from civil society.” [6]

## Participatory governance: country experiences

This section will describe literature on participatory governance experiences in Brazil and Venezuela. The section will analyze country experiences historically mainly along social and political contexts specifically highlighting the state-society dynamics. The idea is to demonstrate how their peculiar context determined the nature and trajectory of participatory governance initiatives. Historically, these countries have been similar in their development/income status, ranked as middle-income countries. Most such countries, and other lower-income countries, are different from high-income countries in that they face much deeper social inequalities as a result of uneven capitalist development, divisive social cleavages based on class, race, or caste, and stiff state bureaucracy based in colonial institutions that thwart working class interests to be represented in the state [5], limiting the scope and depth of democratic deepening [30, 31]. While Brazilian regimes would change hands between elected officials and the military and Venezuelan regimes experienced uninterrupted rule by elected officials, both countries were considered politically stable until the 1960s and the 1970s [32, 33] despite extreme **social exclusion**. Social exclusion in Brazil and Venezuela was in terms of the worst distribution of wealth, not different from the rest of the continent experiencing dissatisfaction with elite rule, and exclusionary political projects that caused or worsened ethnic or economic marginalization [20]. With respect to the **economy,** a close alliance between the state and an emerging national bourgeoisie benefited a small urban middle class but also groups that were socially privileged along race, class, or on other social categories of privilege in their respective contexts [32, 33].

### Brazil

Despite its historical experience of limited democracy that featured political exclusion of the majority, the trajectory of Brazilian democracy post 1970s has steadily achieved greater degree of democratic consolidation with the formation of a relatively autonomous civil society that can effectively engage the state. With the military’s withdrawal from Brazilian politics in the mid-1980s, Brazil had gradually moved from a country with low levels of citizen participation and mobilization, until the early 1980s, to a country known for its participatory institutions [34–37]. The earlier era signified a classical case of political party formation among elites and unstable political system [38]. The country’s early democratization process between 1974 and 1988 had two distinct aspects to it, the reestablishment of political competition, and from the ground-up an increase in voluntary and independent forms of association [39].

The societal aspect of increasing voluntary associations was remarkable given that until the late 1970s very few such associations existed as most citizens were not organized and neither participated in such voluntary associations [40]. This began to change in early 1980s when a large part of Brazilian poor joined participatory institutions [41, 42]. On the political society side of things, prior to the 1980s’ party reform, the country’s institutionalized political system was not great even from Latin American perspective [43]. The creation of a workers’ party *Partido Trabhaldores* (PT) as a mass party with grassroots support began to change this institutionalized political system [44]. The democratic developments in the political society and the societal changes through civil society/social movements increasing their ranks through citizen participation entangled nicely together. PT’s vision of the state aimed towards its subordination to labour and social movements [45, 46]. This vision was aligned with claims of autonomy from the state made by new unionism [47, 48] and the Catholic Church [49]. Autonomy from the state for new unionism meant breaking away from Ministry of Labour’s control of trade union organization, established in 1932, while for the Catholic Church it meant that social actors are able to claim public goods, social services and healthcare, independent of the state [38].

Brazil’s achievements in the health sector have been parallel to the democratic changes going on in the society. Historically, Brazil state-sponsored health tied access to healthcare to work in the formal labour market, a system that dates back to the 1930s and 40s [50]. Brazil was considered to have one of the worst healthcare system in the developing world and access to state-sponsored healthcare was not a universal right until 1988. After 1964, in particular, as the democratic order was broken by a military coup, the healthcare system deteriorated further [46]. Cohn [51] and Elias and Cohn [52] describe these changes in the health sector and how they linked with the democratic changes that were occurring in Brazil starting in the 1970s. Historically, the nature of Brazil’s health care system was bifurcated between a predominantly private system called the Social Prevention system based on the style of social security system and a mainly public health system funded by the state or through philanthropic contributions. The Social Prevention system had its origins in the 1920s and was mainly linked to wage labourer contributions which formed a very small part of the formal and mainly urban-based labour force. This system was highly privatized. Thus, the concept of health as a right was limited to paid contributions by workers and was not seen as a universal right [51]. State provision of health services was limited to public health prevention programs such as vaccination and other health-related activities such as health education and health promotion. No individualized health care was provided to those living in rural areas or working in the informal sector which formed much of the population.

This segmented healthcare system began to change starting in the 1970s and coincided with the democratic changes that were taking place against authoritarian regimes [33, 38]. Just as the Brazilian health care system prior to the 1970s reflected the undemocratic characteristics in the social and political arena of the country, the changes to health care system to a more democratic and participatory one were also occurring parallel to the democratic developments in the political arena [38, 53]. Although during the late 1970s neoliberal ideology was gaining strength internationally, including in the more established social democratic health care systems, democratic changes in the Brazilian health care system were being supported by the country’s political shift to the left. The changes to the health system occurred because of mobilization in health. The health movement in Brazil arose in reaction to measures that the authoritarian regime took regarding the organization of the health system that proved destructive for an already exclusionary health system including: centralizing health and social security system to the federal government but kept the structures of social exclusion; a huge expansion of the private healthcare relative to prevention; significant allocation for building hospitals in large metropolitan areas while diverting funds from local health centers in peripheral and urban areas [38]. The social mobilization around health took the shape of a health movement comprised of representatives from trade unions, health workers, academics, progressive sectors of the Catholic Church and many other civic groups.

The ill condition of Brazil’s healthcare system before democratization with poor quality and lack of access to services prompted two different movements, one professional the *sanitarista* movement (public health professionals’ movement) and the other popular (MOPS) to come together to establish a participatory and inclusive agenda for healthcare reform in democratic Brazil [38]. The *sanitaristas* demanded reform in health through the documentation of inequities in health while linking them to the political struggles [54]. Two important institutions that the *sanitaristas* were instrumental in creating were the Centro Brasileiro de Estudos de Saude (CEBES) and the Associacao Brasileira de Pos-Graduacao em Saude Coletiva (ABRASCO) which served as dissemination of their intellectual analysis at the national level [53]. The *sanitarista* movement emerged in the 1970s as public health professionals were dismayed with the direction of the health systemand to acquire a presence inside the state to shape more inclusionary health policy, instead focused on joining hands with the emerging popular movements where the poor were organizing to claim better access to health care [38]. The *sanitarista* movement went national in 1981 and between 1979 and 1983 at a series of national health conferences the idea of unifying the healthcare system and integrating the newly emerging forms of participation into a national healthcare structure solidified [53]. As described by Cohn [51], the health movement consisted of two important stages. One was the production of knowledge in the health sector that documented inequities in health and linked this knowledge with the political struggle of the time. A discerning element of this knowledge was that it brought an explicitly Marxist perspective in health studies which was also was the result of developments occurring in Latin America in that era. The second stage was the mobilization of organized sections of the society for democratic change in all spheres of life including health. From the 1970s to the 2000s, fertility rates decreased from 5.8 in 1970 to 1.9 in 2000, infant mortality dropped from 114 per 1000 live births to 19.3 per 1000 live births, and life expectancy at birth increased from 52.3 years in 1970 to 72.8 years in 2008 [33].

These developments in health were useful because when the new constitution was being framed towards the end of 1980s, the health sector was at the forefront with alternative proposals for a new health system. The new constitution was passed in 1988 “Article 198 called for a Unified Health System (SUS) that organized a regionalized and decentralized network of health services, with coordinated management at each level of government, community participation, and the prioritizing of prevention as part of an integrated approach to health services delivery.” [52]. The new health system was characterized by universality, decentralized with local autonomy and democratic management under “social control”. The latter was to be achieved mainly through the formation of Health Councils at the national, state, and municipal levels and composed of representatives from both the state and civil society [17, 51]. Health Councils and participatory budgeting are the two most known of the participatory institutions from Brazil, the emergence of their designs with their bottom-up character and enhancing participation in terms of the number of people involved and power-sharing between the state and civil society actors have been key in their growth [38]. During the 1990s, right after the passage of the 1988 Constitution, more than 5000 health councils were formed in Brazil, later growing in number significantly to cover close to 100% of Brazilian cities [55].

Avritzer [38] has in-depth described three main participatory institutional designs in Brazil based mainly on the nature of engagement between civil and political actors. (1) Participatory budgeting is the most bottom-up of the three in which every neighborhood citizen can join and there is deep agreement between the state and civil actors. (2) Health councils comprise power-sharing design that is less participatory than bottom-up and takes place through the election of civil society actors that share decision-making with state actors and are legally institutionalized. (3) Ratification design involved a participatory act following proposal for public policy put forward by the state which was used in city master plans. The eventual origin of participatory institutions in Brazil were based on a bedrock of an emerging social power, in the form of burgeoning civic organizations and neighbourhood associations, that arose either independently or because of repression during authoritarian period while demonstrating social practices demanding accountability, political inclusion, and sovereignty [23]. The success of the new health system also had to do with the fact the social movement had a political strategy as well. The Brazilian Communist Party (PCB) and the Brazilian Workers Party (PT) were both involved in the health movement. At that time many PCB members occupied positions within the state and worked on the inside. PT, on the other hand, had street power as it was a party of the masses and ensured popular mobilizations that worked from the outside. Thus, while popular support was achieved by PT, the communists worked to bring about institutional changes within the state. While the PT was a key actor in participation debates in the healthcare system [38], the defining characteristic of the Brazilian health sector reform was driven by civil society [33]. But this democratization of health did not stop there, the theorizing and design in collective health aimed towards democratization of the state and eventually the society as a whole [56].

A crucial point for this newly passed Brazilian constitution and the newly established health system was that during that time the HIV/AIDS epidemic had started to unfold. Heller [31] mentions that the Brazilian state responded by making HIV/AIDS treatment a priority in response to demands from social movements that made HIV/AIDS a human rights issue and called for comprehensive treatment including free access to anti-retro-viral drugs. In fact, “… Brazil’s strategy of universal treatment has widely touted as a model for stemming the AIDS crisis in the developing world.’” [31]. The response to the HIV/AIDS crisis is indicative of the state-society relationship that existed, and it demonstrates how vitally important meaningful democratic participation can be for political decision-making.

### Venezuela

Changes in the health sector were almost parallel to that in the political sphere and reflected the political ideology. During the 1990s the regimes of Perez and Caldera instituted neoliberal changes in the health sector [57] as part of the wider neoliberal shift in the economy. There was fiscal reduction of public health services and the decentralization of health services. These poorly financed and managed health services when decentralized and handed to regional authorities proved inefficient. Consequently, it paved the way for greater privatization of health services and institution. For those health services that remained under public authorities after widespread privatization effort, cost recovery mechanisms were introduced. The result of these changes in health services was that by 1997, 73% of the total health expenditure in Venezuela was private [57]. Privatization of health services was one of the manifestations of neoliberal policies which disproportionately affected the poor. Consequently class-based health disparities were enormous by the late 1990s [58].

This situation started reversing when Hugo Chavez took power and took the following steps in health between 1998 and 2002 which corresponded with the political changes of his initial years in office. First, he reversed what were popularly known as Caldera Laws which had favoured privatization of the health system and “… implemented a variety of strategies to eliminate barriers to health care …” [57]. These included measures such as the implementation of integrated health care, a focus on primary health care centers and on prevention activities thus shifting the emphasis away from curative care. However, the most significant change was to transform the understanding of health from where it was considered as a commodity to be exchanged in the health care market to one where it was a fundamental right to be provided by the state. In that sense, articles in the newly passed Bolivarian constitution that related to health are illustrative [59]. Health is viewed as a fundamental human right that the state is obligated to guarantee (Article 83). The state has a duty to create and manage a universal, integrated public health system providing free services and prioritizing disease prevention and health promotion (Article 84). This public health system must be publicly financed through taxes, and social security with the state regulating both the public and private elements of the system and developing a human resource policy to train professionals for the new system (Article 85).

Efforts to reform the healthcare system in Venezuela during the initial years of Chavez’s government prior to *Barrio Adentro*, the health reform introduced by Chavez, were based on the Latin American Social Medicine (LASM) principles. LASM has long endorsed an approach in health that is collective rather than individual in nature, focused on the political, economic, and social determinants of health, and emphasized attention to factors that produce health inequities [58]. Corresponding with his initial cabinet appointment of people from the Left [60], Chavez appointed two past presidents of LASM in 1999 and 2001 in health. They tried to implement a healthcare system based on the principles of LASM from the start. However, they faced stiff opposition from the Venezuelan Medical Federation that had aligned itself with the traditional political parties that lost power in the 1990s and the private medical sector that was strongly opposed to Chavez’s attempts to reinvent the healthcare system based on his progressive political ideology that advocated for a strong public health approach for the healthcare system [58].

Steps taken by the State in the first phase to reverse the neoliberal trend in health and other social sectors improved access to health care, but popular mobilization demanded that health services be improved [57]. To deliver effectively to this popular demand, ‘Social Missions’ were established in 2003. Social Missions became motors of the process of social change in Venezuela. The health mission, known as *Barrio Adentro*, was the first to be established and has been at the forefront of the missions because of its achievements [61]. The social missions are social programs created as parallel structures either completely outside the scope of government ministries or in collaboration with them. They act to increase community participation to meet the new constitutional obligations more efficiently [62]. The rationale for creating social missions as parallel structures was to overcome the bureaucratic procedures within the ministries to deliver services efficiently. Additionally, the organizational and logistic capabilities of the Armed Forces were harnessed for civil social actions [57].

*Barrio Adentro* is underpinned by the principles of equity, universality, accessibility, solidarity, multisectoral management, cultural sensitivity, participation, and social justice [63]. “The Mission *Barrio Adentro* will have as its objective the implementation and institutional coordination of a Comprehensive Program of Primary Health Care, the encouragement of the social economy, and the transformation of the social, economic, and environmental conditions within communities under a new model of administration based on principles of inter-dependency, coordination, joint responsibility, and cooperation with the active participation of organized communities in the leading role.” (Presidential decree for the creation of Mission *Barrio Adentro* 2004) [61].

The mission of *Barrio Adentro* was to preferentially provide universal coverage of primary health care for 17 million of formerly excluded citizens. By 2006, 31,439 health care workers were working in the primary care network. Of these, 15,356 were Cuban physicians with approximately 13,000 working in popular medical dispensaries or consultation points [63]. Given that each dispensary/health post serves 250–300 families, the spatial coverage estimation, as reported, comes out to above 70% [61]. Preferential targeting of the *barrio* residents means that the health care coverage is much greater than 70% [63].

A household survey of 270 household heads conducted in various rural and urban areas reported that 51.3% of respondents mentioned that a *Barrio Adentro* facility was located within a walking distance of 5 min [58]. There was a three-fold increase in the number of primary care centers between 1998 and 2007. These new centers were equitably distributed and preferentially located in areas with no or inadequate health facilities thus reducing geographic barriers to health services [57]. Residents of poor neighborhoods reported *Barrio Adentro* as ‘their’ program and resisted Ministry of Health’s efforts to exert limited control. They considered such a move by the Ministry as “… meddling on the part of out-of-touch bureaucrats.” Even middle-class Venezuelans who were interviewed reported *Barrio Adentro* to be a program “for the poor.” [58].

### Participatory governance

*Barrio Adentro* has shown improvements in access to healthcare, utilization of health services, and there is also evidence of improvement in certain health indicators [57, 63]. However, *Barrio Adentro* is known for its community participation. Popular participation in *Barrio Adentro* is achieved through the formation of Health Committees through which communities’ exercise primary health care delivery and management [64]. Health committees are mandated to assess health problems in the community, prioritize them, and decide on the main actions that the community should take to address those problems. Operation of health committees is regulated by the Community Councils Law of 6 April 2006. Among other things the law requires health committees to work with other committees such as those in education, land reform, and water committees that are affiliated with Communal Councils. A National Health Committee Coordinating Office regulates the work of the health committees while similar steps are planned for states and municipalities [63].

Health committees are mandated to work with similar committees in other social missions. Coordination of these committees occurs within Communal Councils which are participatory governance structures for public administration within municipalities. Communal councils are modeled on the local participatory budgeting process in Porto Alegre, Brazil and operate at a sub-municipal level (*communidad*) of between 200 to 400 families in the urban areas and 20 families in the rural areas.

Besides committees for social missions and communal councils several other institutions and mechanisms for participatory democracy have been put in place. These include referenda, social audit/compatrol, citizen assemblies, and cooperatives [60] and reflect the government’s commitment to ensure popular participation in all spheres of life. In the workplace, for example, spaces for small enterprises that are co-managed or self-managed by workers have been opened by many local governments and public institutions [65]. These worker cooperatives allow individuals who work in such enterprises to exercise their democratic participation during production and reflect a model of workplace democracy. Worker cooperatives are a means to popular participation in the Venezuelan economy that were recognized in the 1999 constitution (Article 70). In 2001, a Special Law of Cooperative Associations was passed that facilitated the creation of new cooperatives, emphasized the State’s obligation to protect them, and extended tax-exempt status to them [65].

Such popular arenas of popular participation reflect the importance given to participatory governance in Venezuela. In health, for example, popular participation was established in the Bolivarian constitution as a mechanism to ensure that the state enforces health as a social right. The relevant articles on health in the constitution (Articles 83, 84, 85) have the conceptual underpinning of a “… co-responsibility of the triad of state-individual-society in social participation, which enables citizens and individuals to become the main actors in the new society.” [59] Participation is not only seen narrowly as citizens democratically participating in state affairs but as Article 62 in the constitution, which directly relates to participatory democracy, states “… the participation of people in the formation, control, and execution of public matters is the means necessary to accomplish the protagonism that will guarantee their complete development, both as individuals and collectively.” [65].

## Analysis and discussion

This section will comment on the socio-political context of participatory governance, tracing it historically for the two countries using the ‘socialist compass’. The analysis will involve assessment of the trajectory of the country participatory governance experience using the ‘socialist compass’. The socialist compass provides guidelines to assess social empowerment in a variety of socio-political contexts. This comparative country analysis will facilitate making comments about each country’s trajectory of participatory governance more robustly.

### Brazil

Assessing the socio-political changes in Brazil using the “socialist compass” reveals a move towards greater social empowerment. It stands out because the country’s starting point, the era before the 1970s, can be described as “Capitalist State Economic Regulation” characterized by a system with extensive privatization generally and specifically in health. Citizenship was restricted and highly exclusionary. The concept of social rights was understood being related to the formal economy and through that contributing to social security or being tied to other forms of privilege and power. Such a system catered to a minority while excluding a vast majority of the people. In such a system, capital was the main driving force behind the state. State responsiveness was geared towards interests of the markets or businesses at the expense of the majority sentiment. However, with the changes in the post-1970s period, the situation started moving in the direction of “Statist Socialism”. This was especially the case when PT, as a workers’ party with close ties to other social actors, collectively struggled for alternative institutions and provided a political party platform for demands from civic groups [31, 66]. This struggle resulted in PT gaining political power and enacting policies that were inclusive and participatory. As mentioned by Wright [6], the history of Statist Socialism has tended towards authoritarianism if not accompanied with democratic control of the state. Also, democratic control of social power on the state is limited in Statist Socialism if there is only representative democracy. The Brazilian experience demonstrates that because participatory governance institutions, such as citizen committees or councils, were set-up in tandem with a system of representative democracy, it ensured that these institutions served as conduits for citizen voices in state affairs [67]. However, it must be realized that these remarkable democratic changes that have taken place over the last few decades in Brazil are still mired in capitalistic social relations. Therefore, a more appropriate description would be a mix of “Statist Socialism” and “Social Democratic Statist Economic Regulation”. The establishment of councils, such as health councils, with their power sharing institutional design and other governance councils with members representing the state, civil society, and the relevant interest groups [17] also represent a system of “Associational Democracy”. Social empowerment in Associational Democracy, according to Wright [6], is achieved if such councils are internally democratic. This seems to be the case in Brazil. There is extensive evidence [17, 66, 68], for example, about Brazil’s participatory budgeting experience which demonstrates how the marginalized had adequate representation in various arenas of popular participation.

### Venezuela

Analysis of the situation in Venezuela using the “socialist compass” reveals many similarities with Brazil, although the time frame of these changes is shorter. The situation before and during the 1990s can be described as “Capitalistic State Economic Regulation” which is similar to the starting point in Brazil before and during the 1970s. However, given Venezuela’s predominantly oil-based economy, the grip that economic power had on state power was stronger while social power was meager. During the 1990s the state continued to implement neoliberal policies year after year. Consequently, there was an ever-increasing alignment between state power and economic power while social power was sidelined further. With the social power becoming increasingly estranged within the political system, it resorted to effective collective struggle and mobilization that eventually toppled state power. The situation changed drastically when Hugo Chavez came into power. Initially, it seemed that his governmental actions would resemble “Social Democratic Statist Economic Regulation” while his regime reversed the neoliberal policies enacted by his immediate predecessors. However, his democratically elected government charged itself to regulate an economy that was highly deregulated and privatized.

The change however, was quite drastic and a multi-pronged state intervention was initiated to bring about social change. The intent was to enhance the process of social empowerment along many fronts. Thus, when the state demonstrated its close ties to the marginalized and working classes by taking steps to promote their interests while remaining democratically accountable to them, it resembled “Statist Socialism”. When measures were taken to introduce communal councils and various committees for social missions, it reflected “Associational Democracy”. Similarly, worker cooperatives were promoted and incentives were provided to them by the state to facilitate their growth. Promotion of such economic enterprises of democratic participation indicated a move towards establishing a “Cooperative market economy”. The state realized that such worker cooperatives would be at a disadvantage in a predominantly capitalist-based economy. This was so because worker cooperatives are based on principles of cooperation rather than competition. Thus, the state considered its own obligation to protect these worker cooperatives legally and through the provision of tax-exemption [65]. This was done to promote as many cooperatives as possible such that eventually an economy would emerge based on the principles of cooperation that is socialist in character as opposed to one that is competitive, market-based, and capitalistic in nature.

The eventual aim of all these measures was that a democratic and economic model emerges that is closest to what is described as ‘Participatory Socialism’. Participatory socialism, according to Wright [6], reflects a combination of Statist Socialism and Social Economy. In Participatory Socialism there is a greater role of social power compared to that in Statist Socialism and at the same time there is a greater role of state power compared to that in Social Economy. In Venezuela, the state was promoting greater democratization along various fronts. At the economic front this was being done through measures such as developing worker cooperatives. And through various committees and communal councils, greater democratization was being encouraged through popular participation in state affairs. The state itself in Venezuela was relatively strong but it let social power emerge by developing public spaces to serve as avenues for the practice of participatory governance.

## Conclusion and recommendations

The above analysis indicates that both Brazil and Venezuela were on a path to social empowerment. According to Wright [6], the path to social empowerment may be restricted if there is movement along individual pathways of the “socialist compass”. For social empowerment to be meaningful, there needs to significant movement along multiple pathways. As demonstrated from the analysis, this seems to be the case for both Brazil and Venezuela. Additionally, Wright’s three conditions for actualization of social empowerment that is: a vibrant civil society that is coherent enough to shape state power and economic power; presence of institutional mechanisms which facilitate the mobilization of social power along these pathways; and a capacity to counter power opposed to social empowerment, were being fulfilled to various levels in both Brazil and Venezuela. The need for a strong civil society that continues to be autonomous especially in resisting subordination to the state but at the same time demanding inclusion is essential in both countries for the process of social empowerment to be sustainable. These two characteristics of civil society are vital yet challenging in the Latin American context given the history of clientelism [69].

Beyond Latin America, the implications for health care system governance that is inclusive of civil society participation in other lower- and middle-income countries (LMIC) could be similar given that the social cleavages based along class, race, and gender give rise to health and social inequalities. Such social cleavages can pose barriers to the most vulnerable to access societal institutions such as health systems. Health systems as societal institution not only embody existing social inequalities but without an explicit focus on reducing inequalities, can perpetuate them. Inclusive and participatory health systems governance can be key to bring collective voices of the marginalized in discussions to improve the way health services can be organized to reduce barriers that such groups might face in accessing quality health services. In that sense democratic and participatory institutions across sectors, including in health, can be critical in reducing social inequalities.

The recent political turmoil that both countries have been facing demonstrates how this balance at the state-society nexus is delicate and prone to change both internally as well as by conditions external to the national boundaries. Capitalistic societal relations are entrenched deep in local, national and global socio-political contexts and the economic power domain is much stronger and overpowering that can undermine interests represented by social power. Elite capture of the domain of state power is always a threat and elite capture can then be used to buttress the domain of economic power even further while undermining the domain of social power.

In this paper we have analysed participatory governance initiatives in two countries by highlighting the socio-political context in which such initiatives are designed and implemented. Our analysis has made the case that whether it is particular interventions and/or sector reforms, context plays a key role in the way such efforts are designed and implemented. The evidence that we have presented makes the case to support this hypothesis. We have also used evidence to support our assertion that the design and trajectory of sector reforms are linked to the nature of the state-society dynamics. While we make these associations, we recognize the limitation of our analysis that association and causality in particular require more rigorous studies. Another limitation of our analysis is that it is based on two country cases only which may make our assertions less robust. However, given the uniqueness of this analysis, particularly the novelty of this analysis for health, we hope that further studies along these lines can continue to generate further evidence.

In conclusion, we would emphasize that while conducting such analyses, context is given importance but the politics of that context, especially from a historical perspective, is not focused on as much. The critical sources of power within a society and the inter-play of the power dynamics is equally important. Similarly, it must be realized that power is not static but keeps changing. These are areas that need to be critically looked at while analyzing what those participatory governance efforts aim to achieve, whom and to what extent they engage the citizenry in decision-making processes, and whether such efforts are socially empowering in addition to achieving sector-specific gains, such as improvement in health outcomes and reduction of health inequalities.

## Data Availability

Review article using existing and available literature.

## Abbreviations

AIDS: Acquired Immunodeficiency Syndrome
PCB: Brazilian Communist Party
PT: Brazilian Workers Party
HfA: Health for All
HIV: Human Immunodeficiency Virus
LASM: Latin American Social Medicine
WHO: World Health Organization
